# Reversible Cardiomyopathy Induced by Adrenal Insufficiency: A Case Report

**DOI:** 10.7759/cureus.103300

**Published:** 2026-02-09

**Authors:** Nehad M Makki, Amjad A Bugis, Amr E Waly, Ahmed M Elsheikh, Abdulmueez Abdullah A Moohialdin

**Affiliations:** 1 Endocrinology, Dallah Namar Hospital, Riyadh, SAU; 2 Internal Medicine, Saudi German Hospital, Makkah, SAU; 3 Cardiology, Dallah Namar Hospital, Riyadh, SAU; 4 Endocrinology, International Medical Center, Jeddah, SAU

**Keywords:** adrenal insufficiency, cardiomyopathy, endocrine, hypothyroid, panhypopituitarism

## Abstract

Adrenal insufficiency, though rare, can cause serious cardiovascular complications such as reversible cardiomyopathy. We present the case of a 56-year-old woman admitted to the intensive care unit with bradycardia, hypotension, and hypoglycemia, followed by hemodynamic instability and subsequent development of supraventricular tachycardia requiring stabilization. Endocrine assessment performed after stabilization demonstrated panhypopituitarism with radiological evidence of an empty sella, central hypothyroidism, and biochemical findings consistent with central adrenal insufficiency, confirmed by low morning cortisol with low adrenocorticotropic hormone levels. On admission, echocardiography showed biventricular dysfunction with a reduced ejection fraction and severe tricuspid regurgitation. Following initiation of intravenous hydrocortisone and supportive therapy, repeat echocardiography at one month demonstrated significant improvement, with recovery of systolic function and marked reduction in valvular regurgitation, consistent with reversible cardiomyopathy. This case highlights the importance of adrenal insufficiency as a potential cause of unexplained cardiomyopathy. The patient’s cardiac function improved markedly following appropriate endocrine therapy, with normalization of ejection fraction and resolution of valvular abnormalities. These findings underscore the value of early endocrine evaluation and timely management in preventing irreversible cardiac damage and achieving complete functional recovery.

## Introduction

Adrenal insufficiency, a condition characterized by inadequate production of adrenocorticosteroids, poses significant morbidity and mortality risks, particularly among elderly patients [1]. It arises from various etiologies, including autoimmune disorders, infections, and iatrogenic causes, culminating in a deficiency of cortisol and, in some cases, aldosterone [2]. In primary adrenal insufficiency, the adrenal cortex is directly damaged, often due to autoimmune destruction [3]. Addison’s disease, also known as primary adrenal insufficiency, is associated with a decreased production of glucocorticoid and mineralocorticoid hormones from the adrenal cortex [4]. Secondary adrenal insufficiency results from impaired pituitary adrenocorticotropic hormone (ACTH) secretion, while tertiary adrenal insufficiency arises from hypothalamic dysfunction. Adrenal insufficiency can be life-threatening because of vague symptoms, so immediate diagnosis is critical [3,5].

Diagnostic challenges in adrenal insufficiency often stem from nonspecific symptoms, such as fatigue, weakness, and gastrointestinal complaints, which can overlap with other common medical conditions [6]. The insidious onset of adrenal insufficiency often leads to delayed diagnosis and misinterpretation of symptoms, increasing the risk of adrenal crisis, a life-threatening condition marked by hypotension, electrolyte imbalances, and cardiovascular compromise [2]. The intricate interplay between the hypothalamic-pituitary-adrenal axis and cardiovascular function has become increasingly recognized, suggesting a potential link between adrenal insufficiency and the development of reversible cardiomyopathy [7]. The deficiency in steroid hormones can trigger a cascade of physiological disturbances, affecting electrolyte balance, blood pressure, and overall cardiovascular function [8].

Cardiomyopathy, a heterogeneous group of diseases affecting the heart muscle, can manifest in various forms, including dilated, hypertrophic, and restrictive patterns, each with distinct etiologies and hemodynamic consequences [1]. Studies have shed light on the potential for reversible cardiomyopathy in the context of adrenal insufficiency, challenging the traditional view of cardiomyopathy as a chronic and irreversible condition [3].

Pathophysiological mechanisms linking adrenal insufficiency and cardiomyopathy

The intricate relationship between adrenal insufficiency and cardiomyopathy involves multiple interconnected pathways. Cortisol, a key hormone produced by the adrenal glands, plays a crucial role in maintaining cardiovascular homeostasis by regulating vascular tone, cardiac contractility, and inflammation [9]. Inadequate cortisol production in adrenal insufficiency impairs vascular responsiveness to catecholamines, resulting in reduced systemic vascular resistance and hypotension, which in turn contributes to diminished cardiac output [3,8]. This hemodynamic compromise is further exacerbated by intravascular volume depletion due to cortisol deficiency and, in some cases, aldosterone deficiency, leading to impaired preload and reduced cardiac output [2]. Adrenal insufficiency also disrupts electrolyte homeostasis, particularly sodium and potassium balance, which is essential for normal myocardial excitability and contractility; such disturbances may predispose patients to arrhythmias and exacerbate myocardial dysfunction [3]. Although cardiovascular manifestations of adrenal insufficiency are well recognized, they are traditionally described as hypovolemic hypotension, syncope, and arrhythmias that typically resolve with volume resuscitation and glucocorticoid replacement. Isolated ACTH deficiency, a rare cause of secondary adrenal insufficiency, may lead to low or absent cortisol production despite preservation of other pituitary hormones [10].

Previous case studies and reports

The literature contains a limited number of case reports describing the association between adrenal insufficiency and reversible cardiomyopathy. These reports often highlight the rapid improvement in cardiac function following the initiation of glucocorticoid replacement therapy, suggesting a direct link between adrenal hormone deficiency and myocardial dysfunction. Some patients' cardiac abnormalities resolve entirely after hydrocortisone replacement therapy [11]. One case report described a patient who presented with fulminant biventricular heart failure, mental status changes, and acute kidney injury in the setting of newly diagnosed primary adrenal insufficiency [3]. Echocardiography revealed severely reduced left ventricular ejection fraction, and the patient required mechanical ventilation and vasopressor support. Following the administration of stress-dose glucocorticoids, the patient's hemodynamic status improved dramatically, and subsequent echocardiography demonstrated near-complete recovery of left ventricular function. This case highlighted a relatively uncommon presentation of stress-induced cardiomyopathy secondary to adrenal insufficiency, which was resolved after high doses of glucocorticoids [3]. Another case report described a young girl who developed dilated cardiomyopathy as a complication of Addison's disease and exhibited near-complete recovery of cardiac function with glucocorticoid replacement therapy [12]. While these cases provide valuable insights, they also underscore the need for larger, prospective studies to fully elucidate the prevalence, mechanisms, and long-term outcomes of reversible cardiomyopathy in patients with adrenal insufficiency.

Recent case reports suggest that Takotsubo cardiomyopathy, also referred to as stress cardiomyopathy, is characterized by transient cardiac dysfunction and left ventricular apical ballooning [13]. Takotsubo cardiomyopathy has been observed in patients with aneurysmal subarachnoid hemorrhage and status epilepticus and in a case of acute brain hemorrhage [11,13]. It accounts for 1%-2% of patients presenting with the acute coronary syndrome, with the majority of patients being postmenopausal women [14]. There are several case reports of Takotsubo-like syndrome in patients with pheochromocytoma caused by increased catecholamine secretion. It may be reasonable to hypothesize a similar mechanism with the lack of cortisol leading to subsequent cardiomyopathy [14,15]. Given the complexities in adrenal insufficiency, there is a need for further study of the prevalence of this phenomenon, as well as whether a dose-response relationship exists.

This case was presented to highlight an uncommon manifestation of secondary adrenal insufficiency presenting with reversible cardiomyopathy and significant valvular and rhythm abnormalities in the absence of structural heart disease. It underscores the diagnostic challenge posed by overlapping cardiac, neurological, and endocrine features and demonstrates that timely endocrine evaluation and targeted therapy can result in substantial cardiac recovery. This report therefore expands the limited literature on non-crisis-related, secondary adrenal insufficiency-associated cardiomyopathy.

## Case presentation

**Figure 1:**
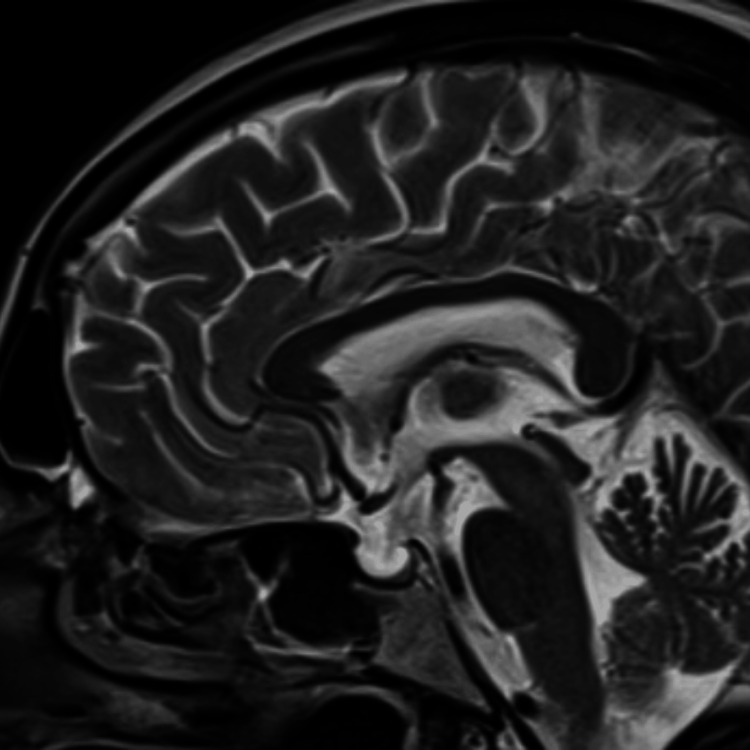
T2-weighted sagittal MRI image of the brain showing an empty sella filled with CSF CSF: cerebrospinal fluid

We present the case of a 56-year-old woman with a history of hypertension (diagnosed in November 2024), treated with bisoprolol 5 mg daily, and long-standing central hypothyroidism (diagnosed 10 years earlier), treated with levothyroxine 125 mcg. The patient was previously admitted on November 28, 2024 (three months back), for a transient ischemic attack and is now taking acetylsalicylic acid. At that time, MRI brain images show multiple focal T2-weighted spin-echo and fluid-attenuated inversion recovery hyperintense signals, suggesting extensive cerebral small vessel disease. There is an empty sella, and the parasailer region is unremarkable (Figure 1). The echocardiography revealed normal left ventricular internal dimensions, systolic function, and grade I diastolic dysfunction. There were no regional wall motion abnormalities. Grade II/IV mitral, tricuspid, and pulmonary regurgitation were present. The right size and function were normal. The ejection fraction was calculated to be 60%.

On March 3, 2025, the patient presented with nausea, vomiting, and hypoglycemia and was immediately transferred to the intensive care unit. She had experienced progressive fatigue, hypotension, and bradycardia over the preceding two weeks. A structured diagnostic evaluation was undertaken, including hormonal assessment, electrocardiography, and transthoracic echocardiography.

Laboratory findings showed hypoglycemia and metabolic acidosis. Echocardiography detected systolic dysfunction, with reduced ejection fraction and severe tricuspid regurgitation. During the hospital stay, she developed supraventricular tachycardia with 180 bpm, and her blood pressure dropped to 70/50 mmHg. The Valsalva maneuver was initiated along with medications like IV metoprolol 5 mg and adenosine 6 mg, but there was no response. Eventually, synchronized cardioversion was performed at 150 J, which resulted in a sinus rhythm with a heart rate of 48 bpm. This condition could potentially be indicative of an adrenal crisis.

Diagnostic evaluation, treatment, and management

Laboratory Analysis

The assessment from the endocrinology side suggests that the patient is suffering from panhypopituitarism in the form of central hypothyroidism and central adrenal insufficiency (Table 1). Although the patient is postmenopausal, she has low follicle-stimulating hormone and luteinizing hormone levels, which are consistent with panhypopituitarism. This is also evidenced by an empty sella on her CT brain scan. The patient was not adherent to the medications during the last four months and revealed an inappropriate normal thyroid-stimulating hormone level despite a low free T4 level. Further, evaluation revealed hypoglycemia, hypotension, hyponatremia, and high-normal potassium levels. This patient has a low morning cortisol level and an ACTH level, which, in conjunction with the patient’s clinical condition, raised suspicion for adrenal insufficiency. These lab results further confirmed the diagnosis of central adrenal insufficiency in this individual. However, the CT adrenal scan revealed a normal adrenal gland with no nodules present.

**Table 1:** Laboratory parameters at the time of admission TSH: thyroid-stimulating hormone; ACTH: adrenocorticotropic hormone

Parameters	Patient values	Reference range
Follicle stimulating hormone	15.11 mIU/mL	Postmenopausal: 23.0-116.3
Luteinizing hormone	9.79 mIU/mL	Postmenopausal: 7.9-53.8
Prolactin	7.01 ng/mL	Postmenopausal: 1.8-20.3
TSH	1.3 uIU/mL	0.55-4.78
Free T4	6.63 pmol/L	10.8-18.7
Free triiodothyronine	3.10 pmol/L	Adults: 3.5-6.5
Cortisol	0.5 ug/dL	Morning serum (7-9 am): 5.27-22.45; afternoon serum (3-5 pm): 3.44-16.76
ACTH	8 pg/mL	10-48

Cardiomyopathy Most Likely Due to Secondary Adrenal Insufficiency

**Figure 2:**
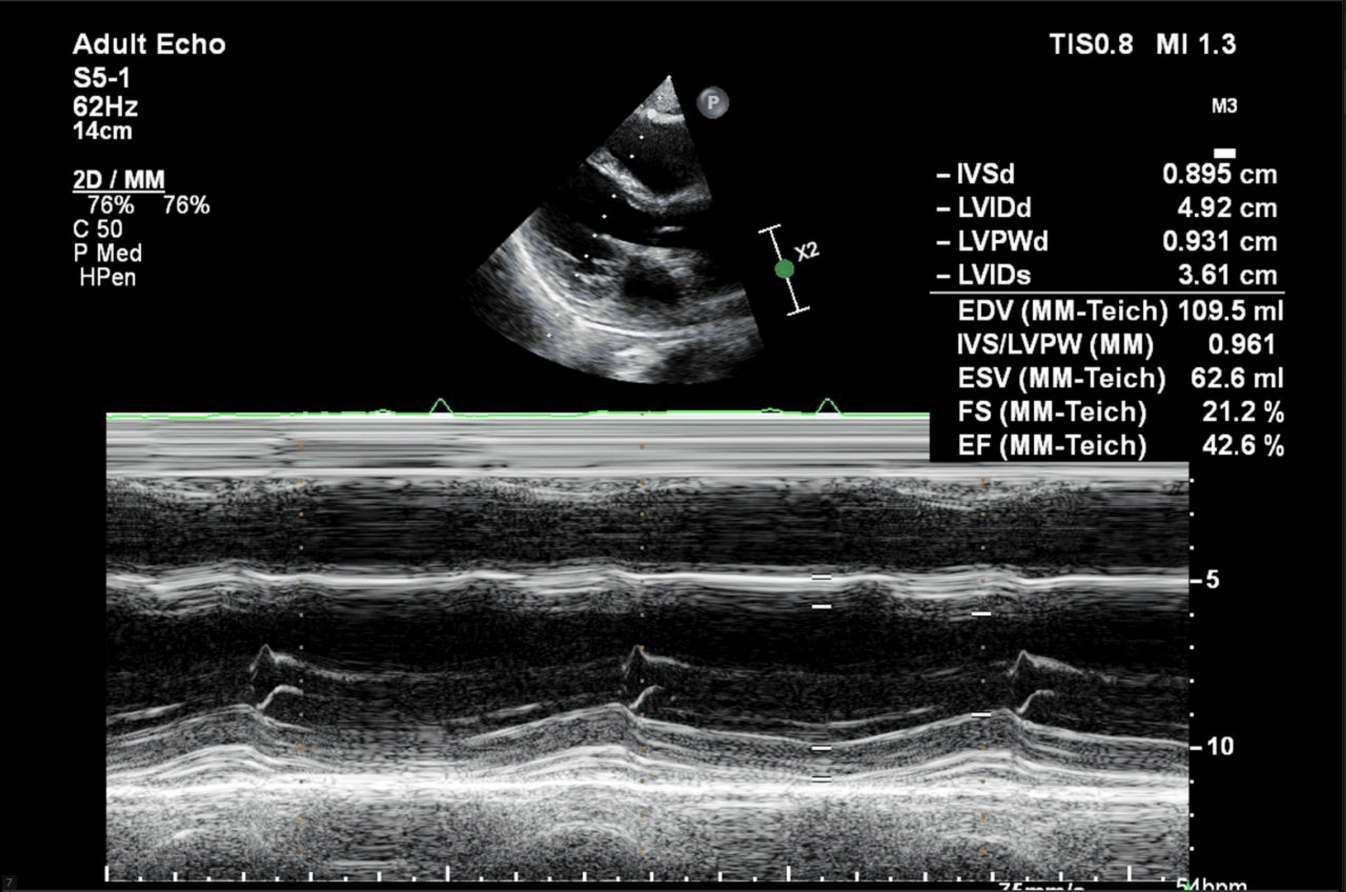
M-mode on LV with estimated function by the Teichholz method with an EF of around 42% (March 25, 2025) LV: left ventricle; IVSd: interventricular septum thickness in end-diastole; LVIDd: left ventricular internal dimension at end-diastole; LVIDs: left ventricular internal dimension at end-systole; LVPWd: left ventricular posterior wall thickness at end-diastole; EDV: end-diastolic volume; IVS: interventricular septum; LVPW: left ventricular posterior wall; ESV: end-systolic volume; FS: fractional shortening; EF: ejection fraction

Echocardiography March 2025: The patient's echocardiogram results reveal a moderately reduced ventricular ejection fraction of around 42%. Additionally, there is Grade 2 left ventricular diastolic dysfunction with a pseudonormal pattern present. The right ventricular cavity size is moderately dilated, and its systolic function is also moderately decreased. There are findings of a mildly dilated left atrium and a moderately dilated right atrium. Mild aortic regurgitation and moderate to severe mitral valve regurgitation are noted, along with severe tricuspid regurgitation and an estimated pulmonary artery systolic pressure of 50 mmHg. A mild pericardial effusion is observed, more prominently anteriorly and at the apex, with less evidence at the basal posterior left ventricle (Figure 2).

Brain Studies

Electroencephalogram: The follow-up electroencephalogram (EEG) was performed under standard conditions with photic stimulation as a provocative maneuver, showing a background mainly composed of low-amplitude delta waves with no clear abnormal epileptic discharge present. This encephalopathic EEG result suggests that there may be some underlying neurological dysfunction or impairment in the brain's electrical activity.

CT brain plain study: A follow-up CT scan shows no interval changes compared with the previous study, except for the resolution of tiny intracranial air loculi. The brainstem, cerebellum, and posterior fossa structures appear normal, with an empty sella noted. There are no significant findings in the visualized orbits or bony settings. However, features of cerebral small vessel disease with suspected tiny lacunar infarcts in the centrum semiovale were observed. There was no evidence of acute infarction, mass effect, or intracranial bleed on the CT scan.

CT Pulmonary Angiography With Multiplanar Reformatted Images

The CT pulmonary angiography revealed pulmonary congestion, atelectasis, emphysematous bullae, small nodules, a prominent atrium, ascites, a retroareolar lesion, and a bulky right thyroid lobe, but no evidence of a pulmonary arterial thromboembolic process.

Treatment

The patient was reviewed by the endocrinology team, and the clinical picture was highly suggestive of adrenal insufficiency, supported by both the presenting features and laboratory findings. Stress-dose glucocorticoid therapy was initiated with intravenous hydrocortisone 100 mg as a bolus, followed by 50 mg every six hours in accordance with standard adrenal insufficiency management protocols, and continued until clinical and biochemical stabilization was achieved. Following clinical and biochemical stabilization, intravenous hydrocortisone was gradually tapered and transitioned to oral physiological replacement dosing, consisting of hydrocortisone 15 mg in the morning and 5 mg in the evening. For presenting signs and symptoms, hypoglycemia was corrected promptly with intravenous dextrose administration. Metabolic acidosis was managed supportively with careful monitoring of electrolytes and fluid status. Hyponatremia was corrected with intravenous NaCl, and hyperkalemia was treated with calcium gluconate, insulin, and salbutamol. For hypothyroidism, levothyroxine was resumed gradually. The patient is currently on a physiological dose of hydrocortisone, taking 15 mg in the morning and 5 mg in the afternoon. Follow-up tests showed normal levels of sodium and potassium, and the patient remains clinically stable. It is recommended to continue with the same dose of hydrocortisone for now.

Discharge plan

The patient was discharged from the hospital after four days, and the follow-up was conducted in the endocrine and cardiology clinic. At the time of discharge, the patient’s blood glucose level was 7 mmol/L. She was hemodynamically stable without inotropic support, with a blood pressure of 110/60 mmHg, heart rate of 76 beats/minute, respiratory rate of 16 breaths/minute, and oxygen saturation 99% on room air, confirming resolution of the initial hypotensive state. Bisoprolol was discontinued, and antihypertensive therapy was switched to perindopril 10 mg once daily along with anticoagulant, levothyroxine, and corticosteroid. An angiotensin-converting enzyme inhibitor was selected due to its proven benefits in patients with left ventricular dysfunction, including reduction in cardiac afterload and preload, improvement in symptoms, and prevention of adverse ventricular remodeling, thereby supporting myocardial recovery.

Follow-up and outcomes

The patient was managed with a multidisciplinary approach, which included endocrinology, cardiology, and neurology expertise that enabled the prompt initiation of hydrocortisone, levothyroxine, electrolyte, anticoagulant, and diuretic therapy, substantially enhancing her hemodynamic stability and overall clinical status. Although residual cardiac dysfunction persisted, there was a partial recovery of left ventricular function at the follow-up.

**Figure 3:**
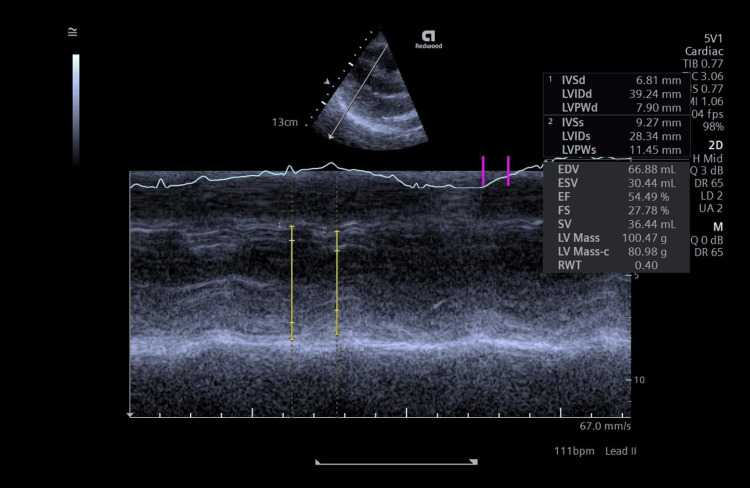
M-mode on LV with estimated function by the Teichholz method, with an EF of around 55% (April 30, 2025) LV: left ventricle; IVSd: interventricular septum thickness in end-diastole; LVIDd: left ventricular internal dimension at end-diastole; LVIDs: left ventricular internal dimension at end-systole; LVPWd: left ventricular posterior wall thickness at end-diastole; EDV: end-diastolic volume; IVSs: interventricular septum thickness in end- systole; LVPW: left ventricular posterior wall; ESV: end-systolic volume; FS: fractional shortening; EF: ejection fraction; SV: stroke volume; RWT: relative wall thickness

After one month, her follow-up assessment shows improved thyroid function, with a free T4 of 16 pmol/L. The patient has normal blood pressure and other vital signs with normal sodium and potassium serum levels. Echocardiography showed improvement in the ejection fraction from 40% to 55% within one month, with improvement of mitral regurgitation from moderate to trace and improvement of tricuspid regurgitation from moderate/severe to mild tricuspid regurgitation (Figure 3).

## Discussion

This case underscores the diagnostic complexities inherent in presentations involving a constellation of cardiac and neurological symptoms, ultimately pointing toward an endocrine etiology. The patient's initial presentation with bradycardia, hypotension, and profound fatigue, coupled with evidence of panhypopituitarism and an empty sella, strongly suggested an underlying endocrine dysregulation rather than primary cardiovascular or neurological pathology [11]. The atypical cardiac findings, including severe tricuspid regurgitation and supraventricular tachycardia, in the absence of atherosclerotic coronary artery disease, further highlighted the systemic rather than localized nature of her illness [11,16].

Alternative etiologies, including myocarditis, ischemic heart disease, primary valvular pathology, and tachycardia-induced cardiomyopathy, were systematically excluded by normal coronary angiography, absence of viral markers or inflammatory changes, and serial echocardiography. Hydrocortisone therapy was initiated on day 1 of ICU admission, after which the patient’s blood pressure normalized, arrhythmias resolved, and ejection fraction improved from 42% to 55% over four weeks. Severe tricuspid regurgitation and mild pulmonary hypertension resolved in parallel, consistent with secondary changes due to ventricular dysfunction. Unlike stress-induced or Takotsubo cardiomyopathy, the patient’s presentation was driven by endocrine deficiency rather than an acute catecholamine surge, highlighting the mechanistic contribution of adrenal insufficiency, electrolyte imbalance, and arrhythmia profile to reversible cardiomyopathy.

This complex interplay of symptoms necessitated a comprehensive diagnostic approach, ultimately revealing adrenal insufficiency as a key contributing factor to the patient's reversible cardiomyopathy and other systemic manifestations [17]. This aligns with existing literature suggesting that endocrine imbalances, particularly those affecting the hypothalamic-pituitary-adrenal axis, can precipitate cardiovascular dysfunction, including cardiomyopathy, through mechanisms involving altered catecholamine sensitivity and fluid-electrolyte disturbances [18].

This case report augments the limited body of evidence regarding reversible cardiomyopathy directly attributable to adrenal insufficiency, particularly when compared to the more commonly reported manifestations like hypovolemic hypotension and arrhythmias [3]. Unlike previous reports that primarily describe acute adrenal crisis presenting with cardiogenic shock [9], our case highlights the potential for cardiomyopathy to develop even in the absence of full-blown shock, emphasizing the need for clinicians to consider adrenal insufficiency in patients with unexplained cardiac dysfunction. Another case details a more insidious onset of biventricular heart failure that improved with steroid administration, similar to other documented cases [19]. This underscores the importance of considering endocrine pathologies in the differential diagnosis of unexplained cardiomyopathy, especially in the absence of conventional cardiovascular risk factors. The transient nature of the cardiac dysfunction observed in this patient, coupled with its resolution following hydrocortisone replacement therapy, further substantiates the direct causal link between adrenal insufficiency and reversible cardiomyopathy, mirroring instances of stress-induced cardiomyopathy that resolve after the inciting stressor is removed [20]. Specifically, the normalization of the QT interval and resolution of ventricular arrhythmias after hydrocortisone treatment observed in previous cases further support the reversibility of cardiac dysfunction associated with adrenal insufficiency [2]. This case also aligns with findings where rapid recovery from refractory heart failure was observed following hydrocortisone replacement therapy in patients with confirmed adrenal insufficiency [11]. This observation reinforces the hypothesis that prompt and appropriate glucocorticoid supplementation can mitigate the deleterious cardiovascular effects of adrenal insufficiency [21]. It is imperative to conduct trials and studies to further explore the association and underlying mechanism of adrenal insufficiency and reversible cardiomyopathy [2,11]. While investigations of adrenal insufficiency in intensive care units have increased, the link between reversible cardiomyopathy and adrenal insufficiency is not well known [2,7].

## Conclusions

Adrenal insufficiency, characterized by a deficiency in glucocorticoid production, can manifest with a spectrum of cardiovascular complications, including reversible cardiomyopathy. At one-month follow-up, our patient demonstrated substantial improvement in cardiac function, with recovery of left ventricular systolic function and a marked reduction in valvular regurgitation; however, longer term follow-up is required to determine the durability of this recovery. While a temporal association between glucocorticoid replacement and cardiac improvement was observed, causality cannot be definitively established based on a single case. This report highlights the importance of maintaining diagnostic vigilance for adrenal insufficiency in patients presenting with unexplained cardiomyopathy, particularly in the critical care setting. Prompt diagnosis and initiation of glucocorticoid replacement therapy can lead to complete restoration of cardiac function. Clinicians should maintain a high index of suspicion for adrenal insufficiency in patients presenting with unexplained cardiomyopathy, particularly in the presence of suggestive signs and symptoms.

Further research is needed to investigate the prevalence, pathophysiology, and long-term outcomes of reversible cardiomyopathy in patients with adrenal insufficiency. Additionally, longitudinal studies are needed to evaluate the durability of cardiac recovery following glucocorticoid replacement therapy and to identify factors that may predict the development of irreversible cardiac damage. Furthermore, investigations into the molecular mechanisms underlying glucocorticoid-mediated cardioprotection may reveal novel therapeutic targets for preventing and treating cardiomyopathy in patients with adrenal insufficiency.

Future research should focus on elucidating the molecular mechanisms underlying glucocorticoid-mediated cardioprotection and identifying novel therapeutic targets for preventing and treating cardiomyopathy in patients with adrenal insufficiency.
